# An innovative application developed to determine the blood output of chickens and its impact on the meat quality in poultry slaughtering

**DOI:** 10.1016/j.psj.2023.103080

**Published:** 2023-08-30

**Authors:** Zeynep Kilci, Ramazan Ulku Cetin, Kivilcim Ates, Didem Tutak

**Affiliations:** ⁎ Department of Food Processing, Agriculture and Forestry Vocational School of Susurluk, Bandirma Onyedi Eylul University, Balikesir, Turkey; † Graduate School of Natural and Applied Sciences, Bursa Uludag University, Bursa, 16059, Turkey; ‡ Research & Development Department, HasTavuk Company, Susurluk, Balikesir, Turkey

**Keywords:** chicken, slaughter innovation, physicochemical quality, microbiological quality

## Abstract

Optimizing blood loss during the slaughtering process is crucial for obtaining high-quality meat, as the presence of meat blood can lead to a reduction in shelf life and negative sensory evaluation by consumers. Moreover, the high water and nutritional content of the meat, along with its appropriate pH value, necessitate careful consideration of the remaining blood, as it can support microbial spoilage of chicken meat. In this context the effects of making an extra cut on conventionally halal-slaughtered broiler's leg which had an extra cut at the cartilage point where the drumstick and the claw part meet, before the bloodletting process were analysed. The results of the analysis indicate that making an additional cut on the drumsticks does not adversely affect the overall quality of chicken meat. Determination of peroxide value (**PV**) and thiobarbituric acid reactive substance (**TBARS**) analyses were performed to analyze the degree of lipid oxidation. The PV and the TBARS value were higher in the drumsticks which have extra cut compared to the uncut samples. L*, a*, and b* values of extra cut thigh meats are higher. However, considering the storage period, the ninth day values in the cut thigh meats were found to be lower than the first day results of the chickens drumsticks do not have an extra cut procedure. As the storage period of chicken drumsticks progressed, as expected, the L* value decreased, while a* value and b* value increased over time. As regards sexes of broilers, it was observed that the *Pseudomonas* spp of female broilers with extra cut in the cartilage tissue of their legs on d 1 was significantly higher than the male broilers. These findings suggest that this innovative method holds significant potential for widespread application.

## INTRODUCTION

In recent years the demand for meat and meat products, which are considered an important protein group of foods, has been constantly increasing worldwide (Wong et al., 2017; Daghir et al., 2021). Today, as consumers become more conscious of food safety and quality, they expect high quality meat (Gonzalez et al., 2020). Meat quality can be affected by a variety of intrinsic and extrinsic factors, including slaughter methods (Bonnet et al., 2020). The animals were manually bled out by severing the carotid arteries and jugular veins in the neck generally (EFSA, 2021). As animals can be exposed to psychological stress during capture in the poultry house and this stress changes the homeostatic balance of the body and directly affects the rate of muscle to meat conversion after slaughter, in a sense, the quality of meat, it is aimed to create freedom of movement for animals in poultry houses. While creating this area, it is tried to prevent stress-related escapes during capture and health problems as a result of collision among animals. However, it is almost impossible to completely eliminate these possibilities in poultry houses with large populations raised for commercial purposes. In addition, the fluttering of the chickens during loading, transport and live hanging and the involvement of external labour in the production process may cause tissue haemorrhages. Since the blood remaining in the carcass generally causes a decrease in the shelf life of the meat and a negative sensory evaluation in consumer preferences, it is necessary to optimize the blood loss during the slaughter process to obtain good quality meat. The amount of blood in poultry is known to be around 6% of liveweight, yet this ratio can vary depending on the age and size of the animal. As the rate and amount of postslaughter bleeding depends on factors such as the anatomy of the animal, the speed at which the animal is slaughtered, the speed of the slaughtering line and the slaughtering process, it is extremely difficult to achieve complete bleeding in practice (Nakyinsige et al., 2014; Anonymous, 2023a; Anonymous, 2023b). In addition, when the water and high nutritional composition of the meat are combined with the appropriate pH value, the blood remaining in the meat stands out as a factor that should be considered to support microbial spoilage (Addeen et al., 2014).

Hemoglobin, a protein rich in iron and found in the blood, is another possible factor that will decrease meat quality by catalyzing lipid oxidation. Once more, in order to eliminate undesirable characteristics including product quality, shelf life, and visual defects, the effectiveness of blood removal during slaughter becomes a crucial issue (Sabow et al., 2016). In countries where people who have embraced the religion of Islam live heavily, paying attention to the “halal slaughter” rules in animal slaughter is an issue that should not be overlooked. For an animal whose meat is halal to eat, to be slaughtered in accordance with religion. (Atasever and Alisarli, 2020). As it can be seen, it is obligatory to bleed the animal thoroughly after slaughter, not only in terms of profit, shelf life, and sensory preferences but also as a practice offered by Muslim consumers.

**Figure 1:**
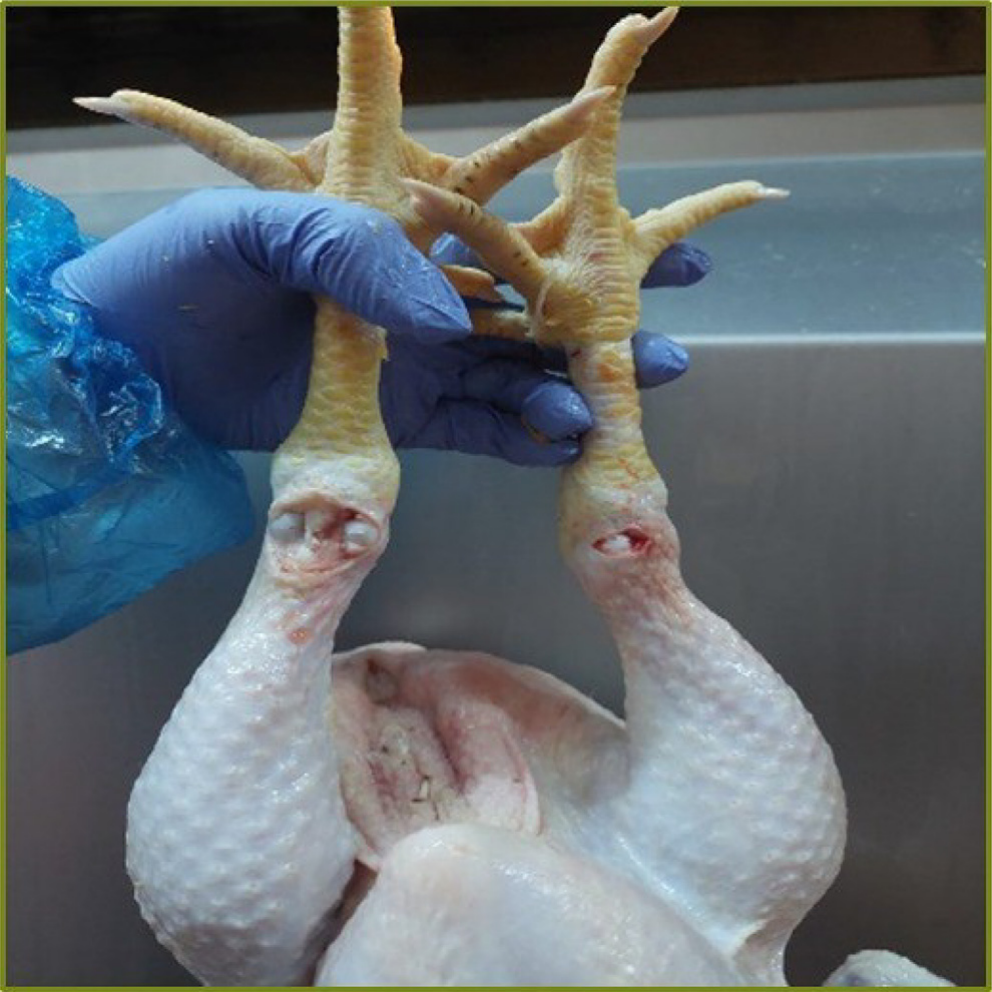
Chicken with extra cut on the cartilage tissue.

**Figure 2:**
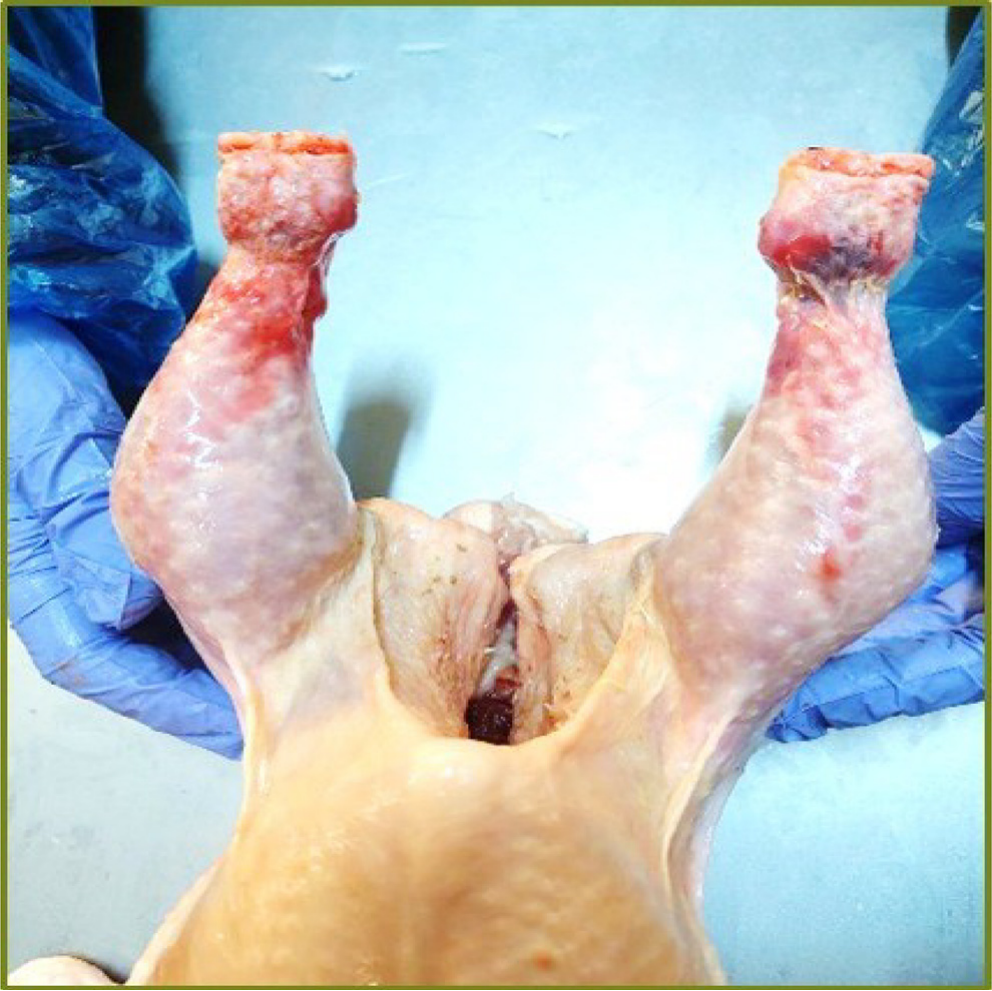
Chicken without extra cut on the cartilage tissue.

It is an indisputable fact that the removal of blood from the slaughtered animal is important in many ways. The effect of the slaughter method on the physicochemical properties of the meat of the slaughtered animal has been studied by many researchers (Gibson et al., 2015; Hassan and Manap, 2015; Ahmed et al., 2018; Zahari et al., 2021). Even it varies between processing plant, the time spent in the blood drainage pool after slaughter is an important issue in terms of both meat quality and product efficiency. The objective is to release approximately 45 to 50% of the total blood present in the chickens into the blood pool. In commercial terms, this corresponds to a target loss of 3 per cent of live body weight. The rate at which live animals suspended by their feet are able to drain their blood by flapping after slaughter is not the equal in all areas. This problem can have a direct impact on meat hygiene and yield (Lopes, 2014). It was predicted that making an extra cut on the thighs of the chickens with a sharp tool before the bloodletting step after slaughtering will have a positive effect on the separation time of blood from the carcass; so some experiments were conducted to determine how much an extra cut in the animal's leg would affect the amount of blood remaining in the tissue. When the average of the trials made for this purpose was taken, it was calculated that while 23.5% blood was observed in the drumsticks which did not have an extra cut, and this rate decreased to 5.8% in the drumsticks which had an extra cut at the cartilage point where the drumstick and the claw part meet. As expected, the presence of blood and/or damaged tissue in the meat causes a decrease in quality. In this sense, while A-quality meat has the closest nutritional and quality characteristics to perfection, it is considered as a subclass B-quality. A decrease in the ratio of B quality meat and an increase in the amount of A-quality products is a positive development that will increase profitability and consumer satisfaction for food companies. Figures 1 and 2 shows images of chicken thighs with and without the innovative cutting process. It can be clearly stated that the meat has a visibly whiter colour after extra cuts are made on the cartilage tissue in the leg. After the chicken is shredded and the thigh part is separated, drumsticks that are likely to be in the A quality group with less blood settlement will be obtained.

In this study, a new solution was developed to minimise the amount of blood remaining in the animal's body, to capture the amount of blood draining from the chest in the drumsticks and to prevent blood remaining in the drumstick. After the butchers perform the halal slaughtering process by manual labour, before the animal enters the blood drainage pool, the extra cutting process is performed at the cartilage point where the drumstick and the claw part meet to prevent blood from remaining on the drumstick. The effects of this extra cut process on the visual quality of the final product, the physico-chemical and microbiological properties of the samples during the postslaughter and storage period were analysed.

Beside an application has been made to TURKPATENT which is affiliated to with the Republic of Turkey Ministry of Industry and Technology regarding this innovative application, which aims to ensure that the blood flows more effectively from the animal by making a cut on the drumstick parts after the slaughtering process of the chickens is completed and before they enter the bloodletting pool. A patent application with reference number PAT-22-0010 with application number e2022/009351 has been filed. The patent process is still ongoing.

## MATERIALS AND METHODS

### Materials

Chicken drumsticks (skin+meat) were obtained from broilers in the same slaughter age range (35 d), with similar weight (average male body weight 2.143 g, average female body weight 2.010 g). In order to determine the effectiveness of the innovative extra cutting process developed, a total of 200 broilers and 400 chicken legs obtained from these broilers were used as samples. these 200 animals consisted of 100 males and 100 females. Among 100 female broilers, 50 were treated with extra cutting on their legs and 50 were not treated with extra cutting on their legs. For 100 male broilers, the procedure was similar; 50 received extra cutting on their legs and 50 did not receive extra cutting on their legs. Totally 400 drumsticks were stored at +4°C for 9 d, and the changes determined by the following analyzes were observed.

### Slaughtering Procedures of The Chickens

First, animals of slaughter age are taken for a health check and confirmed by the veterinarian to be suitable for slaughter. At night, in a dark environment, the animals are gathered in crates without stress and loaded onto live lorries. Animals arriving at the slaughterhouse are held in vehicle holding areas and the first vehicle to arrive is taken to the first slaughter. The crates in the lorry are emptied one by one onto conveyor belts. The belt is used to remove the animals in front of the live hanging personnel from the crates, hold them by the feet and hang them on hangers. The unloading and hanging operations are also carried out in darkness to minimise stress to the animals. Then, electricity is applied to the water in the stun pool and the animals are stunned.

In the slaughter of broilers, electroshock is a method used to make the chicken unconscious and die painlessly. This practice is widely used to reduce stress on the broiler and improve the quality of the meat. Only the beak part of the chicken enters the water while the chicken is upside down, and 240 mA of electricity reaches the brain, preventing the animal from drowning. Once stunned, the animal enters the slaughter line within 15 s.

After the chickens were stunned with the help of electrical energy, they were slaughtered manually by the butchers in accordance with the Islamic methods in accordance with the Halal Slaughter instructions. At this stage, the extra cutting procedure applied to the drumsticks, which is not in the standard flow chart, was performed and then the animals were transferred to the bloodletting pool. Again, in accordance with the halal food criteria, after waiting for at least 180 s of blood to flow, the process continued by going through procedures such as head plucking and feather plucking. Antimicrobial intervention is carried out in the slaughter line with chlorine dioxide (ClO_2_) solution added to the water at a maximum level of 0.5 ppm.

### Physical Analysis of Chicken Meat

#### Moisture Content

The drying trays to be weighed were kept in an oven at 105°C until they reached a constant weight and cooled in a desiccator. The sample was weighed 1.5 g and dried at 105 ± 2°C in a oven until it reached a constant weight. The loss of weight is used to calculate the moisture content of the sample (Cemeroglu, 2010).

$$\%Moisture=\left(m_{2}-m_{3}\right)/\left(m_{2}-m_{1}\right)$$

where *m_1_* represents weight of dried empty drying cup and lid (g), *m_2_* represents weight of drying cup and lid containing test sample before drying (g), and *m_3_* represents weight of sample, cup and lid after drying (g).

### Color Measurement

As recommended by Dadgar et al. (2010), a specific area of chicken meat, free of any obvious color defects, bruises, and blood spots, was analysed by a Hunter Labscan colorimeter (Minolta CR-300, Minolta Corp., Ramsey, NJ). The meat samples were placed on cooled glass plates for the measurements. The *L^∗^, a^∗^,* and *b^∗^* values were recorded for each sample at 3 different points to get a representative evaluation of the samples.

*L*^∗^ is the luminance or lightness component, which ranges from 0 to 100, and parameters *a*^∗^ (from green to red) and *b*^∗^ (from blue to yellow) are the 2 chromatic components, which range from −120 to 120 (Papadakis et al., 2000). In Hunter colorimeter; L* – Lightness coordinate (L*=0 indicates black and L*=100 is white) a* – red/green coordinate, (+a* indicates red, – a* indicates green), b* is the yellow/blue coordinate (+b* indicates yellow and -b* indicates blue).

### Chemical Analysis of Chicken Meat

#### Protein Content

The quantification of raw protein was performed with the AOAC method 960.52 (1995), using micro-digestion and distillation equipment (Foss Tecator 2100 Kjetlec, Hillerød-Denmark), and a conversion factor of 6.25 was used to express the content of raw protein as the % of protein (dry base).

### Total Fat Content

Crude fat content was determined by Soxhlet extraction using petroleum ether as an extraction solvent (AOAC, 2005).

### Ash Content

It was made in accordance with the AACC Method. After the moisture of the crucibles was removed in the oven, they were cooled in the desiccator and 2 g of tared samples were taken into porcelain crucibles and placed in the muffle furnace. It was gradually increased in temperature and burned at 700 ± 5°C until white ash was obtained. After the crucibles were taken into the desiccator and cooled, they were weighed and the results were recorded. Using the weight difference, the results were calculated as % ash content AOAC, 1995).

$$AmountofCrudeAsh\left(\%\right):\left(a_{3}-\;a_{1}\right)/\left(a_{2}\right)$$

where *a_1_* represents weight of porcelain crucible (g), *a_2_* represents weight of sample (g), and *a_3_* represents final weight of porcelain crucible (g).

### Peroxide Value

Primary oxidation products—hydroperoxides - were measured in chicken drumstick samples submitted to the oxidation procedures using both the peroxide value (**PV**) method (AOCS, 1989). Ground sample (1 g) was mixed with 11 mL of chloroform/ methanol (2:1, v/v). The homogenised mixture was then filtered. 2 mL of 0.5% NaCl (w/v) were then added to 7 mL of the filtrate. The mixture was vortexed and then centrifuged to separate the sample into 2 phases. 2 mL of cold chloroform/methanol (2:1, v/v) was added to 3 mL of the lower phase. 25 µL of 30% (w/v) ammonium thiocyanate and 25 μL of 20 mM (w/v) iron (II) chloride were added to the mixture. The reaction mixture was allowed to stand for 20 min at room temperature prior to reading the absorbance at 500 nm. A standard curve was prepared using cumenehydroperoxide at the concentration range of 0.5 to 2 ppm (Addeen et al, 2014).

### Thiobarbituric Acid Reactive Substances

The experiment was followed as being reported by Benjakul and Bauer (2001). Ground-chicken meat (1 g) was added to 5 mL solution (0.375% (w/v) of 2-thiobarbituric acid, 15% (w/v) trichloroacetic acid and 0.25 N (v/v) HCl ). The mixture was incubated in a water bath at 95 °C for 15 min, cooled to room temperature, and later centrifuged at 3,600 r.p.m. at 4°C for 20 min. The supernatant was collected; its absorbance was read at 532 nm using a UV–VIS Spectrophotometer (Varian Cary 50, Varian Inc., California). Thiobarbituric acid reactive substances (**TBARS**) value (expressed as mg malonaldehyde per kg wet sample) was determined from a calibration curve of malonaldehyde standards (2, 4, 6, 8, 10 μmole/L).

### Determination of Mineral Contents

Copper (Cu^+2^), zinc (Zn^+2^), iron (Fe^−2^), manganese (Mn^+2)^, magnesium (Mg^+2^), calcium (Ca^+2^) contents were determined using the Inductively Coupled Plasma Mass Spectrometry (**ICP-MS**) (NMKL, 2001). The collected samples were initially homogenized using blender. Subsequently, the homogenized sample was burned in a microwave device before the analysis in the ICP-MS. 0.2 to 0.5 g of the burned sample was weighed in a vessel. Then, 5 mL of 65% (v/v) Nitric acid (HNO_3_) and 2.5 mL of 50% (v/v) hydrogen peroxide (H_2_O_2_) were pipetted into the sample, respectively. The mixture was then re-burned in microwave device for 70°C/15 min, 85°C/10 min, 105°C/10 min, 110°C/5 min, 120°C/5 min, and finally 130°C/5 min, respectively. After that, the vessel was allowed for cooling in the room conditions for a while. After cooling, the solution in the burned vessel was transferred to a 25 mL flask. Then, deionized water was poured on to it till the final volume in the flask became 25 mL. The analysis of Cu^+2^, Zn^+2^, Fe^+2^, Mn^+2^, Mg^+2^, Ca^+2^ in the samples was performed by using ICP-MS according to the Guidelines of Nordic Committee on Food Analysis (2001) instructions. Before starting the analysis, calibration blank solution, calibration standards, sample blank, control standards, and control samples were all prepared accordingly. Then, a 10 mL of the solution in the 25 mL flask was put into a 10 mL propylen tube. All the solutions were placed in to the ICP- MS device. The reading was started, completed, and the results were automatically processed by the software. The mineral content was calculated and expressed as mg/kg sample.

### Determination of Fatty Acid Composition

Fatty acid methyl esters (**FAMEs**) analysis was performed according to the official methods described in the IOC (2015), respectively. The separation of FAME is performed on analytical columns of specific polarity and length. A flame ionisation detector is used for the detection of the FAME. In a 5 mL screw-top test tube weigh approximately 0.1 g of the sample. Add 2 mL of heptane, and shake. Add 0.2 mL of the 1N (w/v) methanolic potassium hydroxide solution, put on the cap fitted with a joint, tighten the cap, and shake vigorously for 30 s. Leave to stratify until the upper solution becomes clear. Decant the upper layer containing the methyl esters. The heptane solution is ready for injection into the gas chromatograph. It is advisable to keep the solution in the refrigerator until gas chromatographic analysis.

### Microbiological Analysis

Each thigh meat sample (skin+meat) was weighed 10 g into sterile bags, added 90 mL of sterile Ringer's solution and homogenized in a Stomacher device. The microbial load of chicken was enumerated using serial decimal dilutions in the same Ringer's solution and 1 mL of the appropriate dilution was spread on the following growth media: plate count agar, for the determination of total aerobic mesophilic bacteria incubated at 37°C for 24 to 48 h (AOAC, 1990); violet red bile agar, for the determination of total coliform bacteria incubated at 37°C for 24 to 48 h (AOAC, 1994); potato dextrose agar for total mold and dichloran rose-bengal chloramphenicol agar for the determination of total yeast incubated at 25°C for 5 to 7 d (AOAC, 2005); MacConkey agar for the determination of total *Escherichia coli (E. coli)* incubated at 37°C for 24 to 48 h (AOAC, 1994); xylose lactose tergitol 4 agar for the determination of total *Salmonella* spp incubated at 37°C for 24 to 48 h (AOAC, 1984); Baird-Parker agar for the determination of total *Staphylococcus aureus (S. aureus)* incubated at 37°C for 24 to 48 h (Baird-Parker, 1962); *Pseudomonas* Selective Agar Base (supplemented with glycerol) for the determination of total *Pseudomonas* spp incubated at 25°C for 48 h (Goto and Enomoto, 1970). The results were logarithmically transformed and expressed as colony-forming units per gram of sample (cfu/g).

### Statistical Analysis

All experiments were conducted in 2 independent trials, and all trials were performed triplicates. Where appropriate, statistical analyses were conducted using Minitab Release 17. Analysis of variance (ANOVA) and least significant difference test was conducted to determine differences. Significant differences were considered at the 95% confidence level (*P <* 0.05) or at the 99% confidence level (*P <* 0.01).

## RESULTS AND DISCUSSION

### Physicochemical Properties

The ash, total fat, moisture and protein values of both male and female chicken drumsticks which have an extra cut process and do not have the cutting procedure after the normal slaughter operation and the change in these values during the storage period are summarized in Table 1.

**Table 1:** The ash, carbohydrate, fat, moisture, and protein values of chicken drumsticks which were seperated by gender and subjected to an additional cut process during the regular cutting procedure on the storage period (g/100 g).

Samples	Ash (%)	Carbohydrate (%)	Fat (%)	Moisture (%)	Protein (%)
CF1	1.100 ± 0.000^a^	0.010 ± 0.000^cd^	7.443 ± 0.023^a^	74.957 ± 0.012	17.240 ± 0.023^e^
CM1	1.090 ± 0.000^b^	0.010 ± 0.000^cd^	6.783 ± 0.052^b^	74.723 ± 0.079	17.330 ± 0.017^e^
CF9	1.053 ± 0.003^d^	0.030 ± 0.006^ab^	3.183 ± 0.009^e^	77.370 ± 0.290	18.400 ± 0.012^a^
CM9	1.047 ± 0.003^d^	0.030 ± 0.006^ab^	3.850 ± 0.025^d^	76.593 ± 0.058	17.930 ± 0.017^c^
UF1	1.100 ± 0.000^a^	0.000 ± 0.000^d^	7.363 ± 0.027^a^	74.897 ± 0.015	17.500 ± 0.058^d^
UM1	1.070 ± 0.000^c^	0.030 ± 0.006^ab^	6.757 ± 0.007^b^	74.800 ± 0.059	17.300 ± 0.029^e^
UF9	1.067 ± 0.003^c^	0.020 ± 0.000^bc^	4.020 ± 0.006^c^	76.707 ± 0.058	18.170 ± 0.017^b^
UM9	1.033 ± 0.003^e^	0.040 ± 0.000^a^	3.850 ± 0.029^d^	76.803 ± 0.126	17.880 ± 0.006^c^
Statistical Significance	*(*P =* 0.014)	⁎⁎(*P =* 0.003)	⁎⁎(*P =* 0.007)	Ns (*P =* 0.883)	⁎⁎(*P =* 0.005)

Abbreviations: 1, first day of storage; 9, ninth day of storage; CF, Cut&Female chickens’ drumsticks; CM, Cut&Male chickens’ drumsticks; UF, Uncut&Female chickens’ drumsticks; UM, Uncut&Male chickens’ drumsticks.

Values are mean of 3 determinations ± standard error of the mean.

abcde Means in columns followed by different letter(s) are sign significantly different and, ns: indicates not significantly different.

⁎ indicate significant differences at *P < 0*.05;.

⁎⁎ indicate significant differences at *P < 0*.01, respectively.

As can be seen from the table, the moisture values of chicken drumsticks vary between 77.370 and 74.723%, and as expected, the moisture value gradually increased as the storage period progressed. Whether the chicken drumsticks belonged to female or male animals did not cause a significant difference between the moisture values. It can be stated that making an extra cut on chicken drumsticks does not make a significant difference on the moisture value. Meat consists of water, protein, carbohydrates, fat and other dissolved compounds. The moisture content of meat is usually measured between 70% and 80% (Toldra, 2003). In a study conducted by Patriani et al. (2020), it was stated that the moisture value of meat increased to 73.62% after 24 h of storage. In another study, it was stated that the moisture value of chicken meat varied between 70% and 64.7% (Nabievna and Adxamjonovich, 2021). Arizona et al. (2011), stated that the average moisture values of the meats were determined in the range of 76.04 to 76.62%. It can be stated that the amount of moisture in the meat is affected by the feed given to the farm animals and the processes applied to the meat after slaughter, and the observed measurement differences, are due to these situations.

The total protein values of chicken drumsticks vary between 17.240 and 18.400%, total fat values in the range of 3.183 and 7.443%, ash values vary 1.033 and 1.100%, and total carbohydrate values in the range of 0.04-0.01%. It is emphasised that broiler meat contains approximately 71% water, 21% protein, 4% fat and <0.1% carbohydrates in terms of chemical structure (Dawson, 1983). Similarly, it is seen that protein ratio of the chicken drumstick is 16.52%, and the fat value of the raw chicken drumstick is 5% when the study published by Probst (2009) is investigated. In a study searched the effects of animal genotype and nutritional differences on the composition of chicken meat, it was stated that the total ash values in thigh meat samples varied between 0.97 and 1.04% (Meluzzi et al., 2009). In a study conducted on breast and thigh meat of 5 fast, medium and slow-growing chicks, it was determined that there was no significant difference in terms of dry matter or total ash value in meat (skinned and skinless) (Tougan et al., 2013). In a study on yellow Hungarian roosters, it was stated that the total ash content in the thigh meat of chickens was found to be between 0.89 and 0.98% (Konrad and Gaal, 2009). Similar results are detected when literature review for the related analyzes is performed. It can be said that the extra cut, applied to remove the blood from the animal effectively and quickly after slaughter, does not cause a significant change in the nutritional composition of chicken thigh meat in general. Also, it is observed from the data in the table that making an extra cut in the leg at the cartilage point of the animal does not cause a significant difference between the sexes, so it can be stated that it is a procedure easily applied without any concern about the nutritional composition in all broilers.

Peroxide values (PV) and TBARS determinations of both male and female chicken drumsticks which have an extra cut process and do not have the cutting procedure after the normal slaughter operation and the change in these values during the storage period are summarized in Table 2.

**Table 2:** Peroxide values *(PV)* and thiobarbituric acid reactive substances *(TBARS)* of chicken drumsticks which were seperated by gender and subjected to an additional cut process during the regular cutting procedure on the storage period.

Samples	PV (meq O_2_/kg fat)	TBARS (mg MDA/kg of meat)
CF1	0.047 ± 0.003^e^	0.167 ± 0.003^e^
CM1	0.033 ± 0.003^e^	0.203 ± 0.003^e^
CF9	0.830 ± 0.025^a^	1.373 ± 0.035^a^
CM9	0.727 ± 0.018^b^	1.233 ± 0.029^b^
UF1	0.000 ± 0.000^e^	0.003 ± 0.003^f^
UM1	0.020 ± 0.000^e^	0.003 ± 0.003^f^
UF9	0.463 ± 0.009^c^	0.810 ± 0.006^c^
UM9	0.330 ± 0.006^d^	0.713 ± 0.007^d^
Statistical Significance	*(*P =* 0.004)	*(*P =* 0.003)

Abbreviations: 1, first day of storage; 9, ninth day of storage; CF, Cut&Female chickens’ drumsticks; CM, Cut&Male chickens’ drumsticks; PV, peroxide value; TBARS, thiobarbituric acid reactive substances; UF, Uncut&Female chickens’ drumsticks; UM, Uncut&Male chickens’ drumsticks.

Values are mean of 3 determinations ± standard error of the mean.

abcdef Means in columns followed by different letter(s) are sign significantly different.

⁎ indicate significant differences at *P < 0*.01, respectively.

The PV and the TBARS value were higher in the drumsticks which have extra cut at the cartilage point compared to the uncut samples. Regardless of gender, both PV and TBARS increased during storage in chicken thigh with and without the developed method to ensure effective removal of blood from the animal. Lipid oxidation is a deterioration process that causes significant quality losses in meats during storage. It is responsible for a wide variety of undesirable reactions, such as loss of fresh meat color and flavor, while protein oxidation products cause a simultaneous loss of protein functionality and nutritional value (Soyer et al., 2010; Coombs et al., 2018). The main factors affecting lipid oxidation in raw meat include fatty acid composition, endogenous prooxidative or antioxidative components, and nonmeat additives (antioxidative or prooxidative) (Gheisari et al., 2010). Determination of PV and TBARS analyses were performed to analyze the degree of lipid oxidation. Since it is the most important oxidation product in meat and its products, TBARS analysis is performed to determine the amount of malondialdehyde in unit meat (Rasinska et al., 2019). The fact that the samples with high PV and TBARS values are also samples with high unsaturated fatty acids, which supports each other. In addition, the use of metal parts during the cut process in order to ensure effective blood flow should be considered as a factor that increases the possibility of oxidation.

### Color Properties

The color analysis (L*, a*, b*) of both male and female chicken drumsticks which have an extra cut process at the cartilage point and do not have the cutting procedure after the normal slaughter operation and the change in these values during the storage period are summarized in Table 3.

**Table 3:** The L*, a*, b* values of chicken drumsticks which were seperated by gender and subjected to an additional cut process during the regular cutting procedure on the storage period.

Sample	L*	a*	b*
CF1	65.937 ± 0.438^a^	3.310 ± 0.123^e^	8.283 ± 0.148^e^
CM1	61.193 ± 0.047^b^	4.383 ± 0.137^d^	8.983 ± 0.035^d^^e^
CF9	57.243 ± 0.139^d^	5.723 ± 0.050^b^	12.080 ± 0.182^b^
CM9	55.513 ± 0.061^e^	6.163 ± 0.155^a^^b^	11.660 ± 0.182^b^
UF1	59.823 ± 0.088^c^	4.473 ± 0.066^d^	10.667 ± 0.221^c^
UM1	57.393 ± 0.378^d^	5.000 ± 0.163^c^	9.703 ± 0.144^d^
UF9	53.440 ± 0.225^f^	6.417 ± 0.133^a^	13.373 ± 0.245^a^
UM9	52.113 ± 0.052^g^	6.393 ± 0.135^a^	13.917 ± 0.295^a^
Statistical Significance	*(*P =* 0.007)	*(*P =* 0.009)	*(*P =* 0.002)

Abbreviations: 1, first day of storage; 9, ninth day of storage; CF, Cut&Female chickens’ drumsticks; CM, Cut&Male chickens’ drumsticks; UF, Uncut&Female chickens’ drumsticks; UM, Uncut&Male chickens’ drumsticks.

Values are from average of 3 determinations ± standard error of the mean

abcdefg Means in columns followed by different letter(s) are sign significantly different.

⁎ indicate significant differences at *P < 0*.01, respectively.

As can be seen from the table, L*, a* and b* values of extra cut thigh meats are higher. These results can be seen as a kind of proof that the extra cut on the cartilage tissue performs well in terms of blood drainage and having less damaged tissue. However, considering the storage period, the 9th day values in the cut thigh meats were found to be lower than the 1st day results of the chickens drumsticks do not have an extra cut procedure. As the storage period of chicken drumsticks progressed, as expected, the L* value decreased, while a* value and b* value increased over time. Since cutting the drumsticks helps the blood to flow more from the meat, higher L* value (Cut&Female drumsticks on first day: 65.937, Cut&Male drumsticks on first day: 61.193b Cut&Female drumsticks on ninth day: 57.243, Cut&Male drumsticks on ninth day: 55.513) and lower a* values (Cut&Female drumsticks on first day 3.310, Cut&Male drumsticks on first Day 4.383, Cut&Female drumsticks on ninth day 5.723, Cut&Male drumsticks on ninth day 6.163) are seen in the drumsticks which had extra cut. The effect of dulling on the surfaces of the stored meat over time is also supported by the increase in yellow color (b*). The effect of cooling methods on color investigated and L*, a*, and b* values found similar to the uncut thigh samples in our study were detected in the analyzed chicken thighs (Wang et al., 2020). Khare et al. (2014), it was stated that similar conversion were experienced in the color values of chicken meat samples during storage.

### Nutritional Properties

The mineral content of both male and female chicken drumsticks which have an extra cut process and do not have the cutting procedure after the normal slaughter operation and the change in these values during the storage period are summarized in Table 4.

**Table 4:** Mineral content of chicken drumsticks which were eparated by gender and subjected to an additional cut process during the regular cutting procedure on the storage period (g/100 g).

Sample	Cu^+2^	Zn^+2^	Fe^−2^	Mn^+2^	Mg^+2^	Ca^+2^
CF1	0.34 ± 0.14	21.73 ± 0.87	6.79 ± 1.15	ND	143.62 ± 27.29	245.62 ± 39.30
CM1	0.33 ± 0.12	17.32 ± 0.69	7.86 ± 1.34	ND	176.77 ± 33.59	272.98 ± 43.68
CF9	0.34 ± 0.04	19.11 ± 0.76	5.33 ± 0.91	ND	260.07 ± 49.41	94.63 ± 15.94
CM9	0.33 ± 0.13	15.42 ± 0.89	6.01 ± 1.02	ND	286.96 ± 25.27	106.32 ± 38.42
UF1	0.33 ± 0.08	23.06 ± 0.92	8.59 ± 1.46	ND	164.32 ± 31.22	252.32 ± 104.37
UM1	0.29 ± 0.11	16.61 ± 0.66	7.94 ± 1.35	ND	171.39 ± 32.56	272.03 ± 43.52
UF9	0.32 ± 0.05	19.88 ± 0.80	6.60 ± 1.12	ND	251.42 ± 47.77	99.41 ± 15.27
UM9	0.28 ± 0.14	14.81 ± 0.46	6.76 ± 1.17	ND	281.87 ± 29.34	105.43 ± 24.54

Abbreviations: 1, first day of storage; 9, ninth day of storage; Ca^+2^, Calcium; CF, Cut&Female chickens’ drumsticks; CM, Cut&Male chickens’ drumsticks; Cu^+2^, copper; Fe^−2^, iron; Mg^+2^, magnesium; Mn^+2^, manganese; ND, not detectable or below detection limit; UF, Uncut&Female chickens’ drumsticks; UM, Uncut&Male chickens’ drumsticks; Zn^+2^, zinc.

Values are mean of 3 determinations ± standard error of the mean.

There is slightly noticeable difference between the samples in terms of Cu^+2^, Zn^+2^, Fe^−2^, Mn^+2^, Mg^+2^, and Ca^+2^ when the impact of the cutting procedure applied in addition to the standard slaughtering operation on the mineral composition of the thigh meat is evaluated. Whereas the amount of Mg^+2^ increases significantly over the course of the 9-d storage period, the Ca^+2^ mineral shows the reverse trend, declining. The high amount of Ca^+2^ and Mg^+2^ minerals measured in chicken thigh meat samples by Adeen et al. (2014) is also supported by the study. Fe^−2^ and Cu^+2^ act as pro-oxidant in chicken meat during storage. Among the transition metal ions, especially Cu^+2^ and Fe^−2^ are known as the main catalysts of oxidation (Thanonkaew et al., 2006). The decrease in Fe^−2^ amount over time and the PV, which is an indicator of oxidation, coincide with the increase in TBARS and support each other. The fact that the amount of Fe^−2^ is detected in lower amounts in cut thigh meat can be considered as an indicator of effective removal of blood from the meat. It is seen in the table that the amount of Zn^+2^ mineral is relatively higher in female broilers, while the amount of Fe^−2^, Ca^+2^, and Mg^+2^ is higher in male broilers.

The fatty acid profiles of both male and female chicken drumsticks which have an extra cut process and do not have the cutting procedure after the normal slaughter operation and the change in these values during the storage period are summarized in Table 5.

**Table 5:** The fatty acid profile of chicken drumsticks which were seperated by gender and subjected to an additional cut process during the regular cutting procedure on the storage period (g/100 g).

Fatty acid	CF1	CM1	CF9	CM9	UF1	UM1	UF9	UM9
Caproic acid	0.020	0.020	ND	ND	0.010	0.020	0.010	0.010
Caprilic acid	0.060	0.060	ND	ND	0.070	0.070	0.020	0.030
Myristic acid	0.060	0.050	0.040	0.030	0.080	0.060	0.040	0.030
Myristoleic acid	0.010	0.010	ND	ND	ND	0.010	ND	ND
Pentadecanoic acid	0.010	0.010	ND	ND	ND	0.010	ND	ND
Palmitic acid	2.580	2.350	1.500	1.350	2.890	2.800	1.570	1.510
Palmitoleic acid	0.270	0.260	0.070	0.070	0.280	0.230	0.100	0.200
Margaric acid	0.030	0.030	0.010	0.010	0.030	0.030	0.020	0.020
Heptadecenoic acid	0.040	0.040	0.010	0.010	0.030	0.030	0.020	0.020
Stearic acid	0.810	0.720	0.510	0.550	0.910	0.880	0.550	0.610
Oleic acid	2.940	2.740	0.960	0.720	2.820	2.540	1.450	1.330
Linoleic acid	3.080	2.870	1.570	1.410	3.110	2.970	1.790	1.680
Alpha-linolenic acid	0.010	0.010	ND	ND	ND	ND	ND	ND
Linolenic acid	0.010	ND	ND	ND	ND	ND	ND	ND
Gadoleic acid	0.030	0.030	0.010	0.020	0.020	0.020	0.010	0.010
Saturated fatty acid	3.600	3.260	2.070	1.920	4.020	3.900	2.200	1.700
Unsaturated fatty acids	3.790	3.620	1.120	1.000	3.360	3.000	1.820	1.010
Mono-unsaturated fatty acids	3.290	3.080	1.050	0.940	3.140	2.830	1.580	0.830
Poly-unsaturated fatty acids	0.500	0.540	0.070	0.060	0.220	0.170	0.230	0.180

Abbreviations: 1, first day of storage; 9, ninth day of storage; CF, Cut&Female chickens’ drumsticks; CM, Cut&Male chickens’ drumsticks; UF, Uncut&Female chickens’ drumsticks; UM, Uncut&Male chickens’ drumsticks.

Values are are mean of 3 determinations.

The major fatty acids in chicken meat, according to an analysis of their fatty acid profiles, are oleic acid and linoleic acid as well as palmitic acid. Unsaturated fatty acids are sensitive to oxidation and oxidize in long-term storage. As a matter of fact, the decrease in the amount of polyunsaturated fatty acids in the analyzes performed on the 9th day of storage proves this oxidation state. TBARS analyzes with increasing PV during storage also support the oxidation state. In a study conducted on sea bass and red tilapia fish, it was determined that triglycerides and phospholipids were broken down into free fatty acids that are sensitive to oxidation during storage (Thiansilakul et al., 2010). In a study searching the effect of different slaughtering methods on the quality characteristics of chicken meat, it was determined that the main components of the lipid content of chicken meat are mono and polyunsaturated fatty acids (Addeen et al., 2014). The fatty acid profile of chicken breast and thigh, it was stated that unsaturated fatty acids were dominant and a profile rich in monosaturated fatty acids was determined in another research (Cortinas et al., 2004). Once more, in a researchers looked at the fatty acid profile of chicken meat, it was shown that mono and polyunsaturated fatty acids had the highest percentage in total, with oleic acid and linoleic acid being the dominating fatty acids in this ratio with palmitic acid (De Marchi et al., 2012). In a study conducted to determine the fatty acid compositions of some frequently consumed foods in Turkey, similarly, the dominant fatty acids in chicken meat were oleic acid, followed by linoleic acid and palmitic acid (Karabulut, 2009). Boz et al. (2021), it was stated that a similar profile was obtained as dominant fatty acids in both breast meat and thigh meat. Considering the fatty acid distribution in terms of sex, it can be stated that there are no significant differences between male and female broilers, and the quantitative difference is observed more clearly depending on time.

### Microbiological Properties

The results of microbiological analysis of both male and female chicken drumsticks which have an extra cut process and do not have the cutting procedure after the normal slaughter operation and the change in these values during the storage period are summarized in Table 6.

**Table 6:** Microbiological analysis results of chicken drumsticks which were seperated by gender and subjected to an additional cut process during the regular cutting procedure on the storage period (log_10_ cfu/g).

Sample	TMAB	Coliform	Yeast	Mold	*E. coli*	Salmonella spp	*S. aureus*	Pseudomonas spp
CF1	3.903 ± 0.015^f^	0.000 ± 0.000^c^	0.000 ± 0.000	0.000 ± 0.000^c^	0.000 ± 0.000	0.000 ± 0.000	2.760 ± 0.006^fg^	2.847 ± 0.009^d^
CM1	3.823 ± 0.003^g^	0.000 ± 0.000^c^	0.000 ± 0.000	0.000 ± 0.000^c^	0.000 ± 0.000	0.000 ± 0.000	2.717 ± 0.003^g^	0.000 ± 0.000^f^
CF9	5.190 ± 0.006^c^	0.000 ± 0.000^c^	1.977 ± 0.015	1.000 ± 0.000^a^	0.000 ± 0.000	0.000 ± 0.000	3.333 ± 0.003^d^	4.377 ± 0.003^c^
CM9	5.210 ± 0.010^c^	0.280 ± 0.020^b^	2.077 ± 0.020	0.867 ± 0.017^b^	0.100 ± 0.100	0.000 ± 0.000	3.430 ± 0.012^c^	4.407 ± 0.061^c^
UF1	4.013 ± 0.013^e^	0.000 ± 0.000^c^	0.333 ± 0.333	0.000 ± 0.000^c^	0.000 ± 0.000	0.000 ± 0.000	2.883 ± 0.003^e^	2.927 ± 0.015^d^
UM1	4.380 ± 0.012^d^	0.000 ± 0.000^c^	0.433 ± 0.433	0.000 ± 0.000^c^	0.000 ± 0.000	0.000 ± 0.000	2.820 ± 0.000^ef^	2.570 ± 0.017^e^
UF9	5.520 ± 0.006^a^	0.333 ± 0.033^ab^	2.100 ± 0.032	0.967 ± 0.017^a^	0.300 ± 0.300	0.000 ± 0.000	4.200 ± 0.012^a^	4.870 ± 0.006^a^
UM9	5.457 ± 0.003^b^	0.420 ± 0.060^a^	2.440 ± 0.058	0.967 ± 0.167^a^	0.000 ± 0.000	0.000 ± 0.000	4.040 ± 0.023^b^	4.620 ± 0.023^b^
Statistical Significance	*(*P =* 0.002)	*(*P =* 0.002)	Ns (*P =* 0.896)	*(*P =* 0.009)	Ns (*P =* 0.508)	ns	*(*P =* 0.003)	*(*P =* 0.002)

Abbreviations: 1, first day of storage; 9, ninth day of storage; CF, Cut&Female; CM, Cut&Male; *E. coli, Escherichia coli; S. aureus, Staphylococcus aureus*; TMAB, total mesophilic bacteria; UF, Uncut&Female; UM, Uncut&Male chickens’ drumsticks*.*

Values are mean of 3 determinations ± standard error of the mean.

abcdefg Means in columns followed by different letter(s) are sign significantly different and, ns: indicates not significantly different

⁎ Indicate significant differences at *P* < 0.01, respectively.

As seen in the tables, total mesophilic aerobic bacteria (**TMAB**; 3.823–4.380 log_10_ cfu/g), *S. aureus* (2.717–2.883 log_10_ cfu/g), and *Pseudomonas* spp (2.570–2.927 log_10_ cfu/g) were detected in chicken thigh meat samples on d 1. Standard slaughtering procedure was applied and yeast growth was observed on first day (0.333 and 0.433 log_10_ cfu/g) in drumstick samples without extra cuts. Coliform bacteria, total mold, and *E.coli* growth were not observed. When the storage process was continued, it was determined that the microbial load increased in all microbial analyzes, while *Salmonella* spp was not observed even on the 9th day. When the [Bibr bib0073][Bibr bib0073] is examined, it is seen that the limit values for the relevant microorganisms are not exceeded.

Total mesophilic aerobic bacteria (TMAB) count is an important criterion that provides information about processing and storage conditions, product safety and product quality in meat and meat products (Bell, 2002). The TMAB count was found to be lower both on the first day and on the ninth day in the extra cut thigh meat samples in order to ensure the effective removal of blood after the animal was slaughtered. It is evaluated that the microbial load is detected lower in thigh meat samples with the effective removal of blood. The values found in researches carried out in a comparable setting demonstrate consistency with current study (Eglezos et al., 2008; Al-Dughaym et al., 2010; Scheinberg et al., 2013; Murad et al., 2014). As a result of the microbiological analyses of male and female broilers, it can be stated that an increase in the number of TMAB was observed with respect to time rather than sex.

The detection of an insignificant level of coliform bacteria and *E. coli* in the samples analyzed within the scope of our study, even on the ninth day of storage, shows that the production and storage conditions are suitable for hygienic measures. The presence of *Salmonella* spp was not detected in the samples analyzed within the scope of our study, even on the last day of storage.

*Salmonella* spp. not being found even on the final day of the 9-d storage period puts the samples in a much better position than the maximum limits permitted by the Turkish Food Codex Microbiological Criteria Regulation (2011) when  general evaluation of the coliform bacteria, *E. coli*, and *Salmonella* spp. numbers in the samples is desired. When measured in terms of the presence of *E. coli* and coliform bacteria, it was found that the presence of microorganisms was within the permitted ranges under the applicable Regulation. It is possible to conclude that the samples reflect good hygiene, sanitation, and food safety standards. It is seen in the table that lower numbers are measured in the samples with extra cuts applied to remove the blood from the slaughtered animal more effectively and quickly among the samples in which the presence of microorganisms is detected. At this point, it can be evaluated that as the rate of removal of blood from the animal increases, a higher quality product is obtained in terms of microbial. Coliform bacteria, *E. coli* and the *Salmonella* genus in the *Enterobacteriaceae* family are facultative anaerobic, gram-negative, nonspore forming, rod-shaped bacteria that form gas and acid from lactose in 48 h at 35°C to 37°C. Since coliform bacteria and *E. coli* are common both in the intestine and in nature (soil, plant, etc.), they are considered sanitation indicator in the food industry. Therefore, the presence of high levels of these microorganisms in meat and meat products is accepted as an indication that the necessary hygienic measures are not taken during or after slaughter or during production, storage and sale (Jay et al., 2008; Abatcha et al., 2018). Some strains of *E. coli* cause gastrointestinal, urinary tract and central nervous system diseases and septicemia. Pathogenic types of *E. coli* strains can cause diseases such as diarrhea, wound infections, meningitis, septicemia, arteriosclerosis, hemolytic uremic syndrome, etc. Foods at risk for *E. coli* strains are raw or untreated meat and meat products, milk and dairy products, and apple juice (Bell, 2002; Cooley et al., 2007). *Salmonella*, a classical food infection known as salmonellosis, is of great importance in food microbiology. Gastroenteritis caused by *Salmonella* can result in death. Even if there is a very low level of *Salmonella* in foods, *Salmonella* is not allowed in foodstuffs, drinking and utility water as they are considered risky. Since it is commonly seen in animals, especially poultry, animal products are the most common foods. Inappropriate raw materials, processing technology, storage and marketing conditions can increase the risk of *Salmonella* (Rivera-Pérez et al., 2014).

Molds and yeasts are another group of microorganisms that are effective in spoiling chicken meat. Yeast and molds can grow in a wide pH range (pH 2–9), storage temperature (10°C–35°C) and water activity (0.85 and above). In addition, they can use the protein, lipids, organic acids and other complex carbohydrates (Mossel et al., 1962; Efe and Gumussoy 2005). While no mold growth was observed in the samples on the first day, yeast growth was observed (0.333 and 0.433 log_10_ cfu/g) in the samples that were not cut. When the results of the ninth day were examined, yeast and mold growth were observed in all samples. If the storage process is continued, the number of yeasts and molds will continue to increase and the product will weaken day by day in terms of nutritional composition and eventually deteriorate. Although the microbial load was detected at a lower level in the samples with have extra cut procedure, the difference between the samples was not determined at a significant level. Researches with higher yeast and mold counts compared to our samples, even on the first day, in microbiological analyzes of chicken meat (Cetin, 2006;Kozacinski et al., 2006; Yildirim et al., 2015) are available in the literature. In this respect, it can be claimed that all male and female samples, with or without extra cuts in the cartilage tissue of their legs, are of good quality in terms of microbial load.

*S. aureus* has many potential virulence factors, these are; various surface proteins, phagocytosis-inhibiting factors, and toxins that damage tissues and cause disease symptoms. *S. aureus*, one of the most common foodborne pathogens worldwide, is an important pathogen causing many food poisoning and nosocomial infections (Bhunia, 2018). Food contamination with *S. aureus* results from insufficient sanitary conditions during food processing or from food obtained directly from infected animals. Meat and meat products produced under inappropriate conditions and kept in warehouses for a long time before being put on the market pose a significant risk to human health in terms of the presence of pathogenic organisms and enterotoxins. (Thapaliya et al., 2017). *S. aureus* was detected in all samples on both d 1 and d 9. However, as seen in the other microbiological analyzes, less *S. aureus* was detected in the samples with extra cutting process. *S. aureus* analysis was performed and a higher presence of microorganisms was found in similar studies (Sezen, 2009; Yildirim et al., 2015; Sancak et al., 2020). The fact that the permitted limits in the Turkish Food Codex Microbiological Criteria Regulation (2011) were not exceeded also showed that the samples were of good quality for *S. aureus*.

*Pseudomonas* spp is an aerobic, gram-negative bacterium that is commonly found in soil. It can grow well in a range of temperature levels, from 2 to 35°C, and can be easily found in chilled food products, as well as food prepared at room temperature (Ercolini et al., 2010). As research published in the literature with a similar scope were assessed (Sheir et al., 2020; Yu et al., 2020), it was seen that the findings were consistent with the values we reported in our study. As regards sexes of broilers, it was observed that the *Pseudomonas* spp of female broilers with extra cut in the cartilage tissue of their legs on d 1 was significantly higher than the male broilers. It is possible to suspect the contamination of the *Pseudomonas* spp during the application of cutting.

## CONCLUSION

Consumers meet most of their daily animal protein needs from chicken meat, as poultry meat, especially chicken, is economical, has high nutritional value and digestibility, and has low cholesterol and fat content. However, more than 40% of B-quality product separation in chicken meat slaughterhouses is due to carcass quality problems. With the development of technology in order to produce serial and high-quality chicken meat while meeting the supply quantities, enterprises aim to minimize the discharge time of blood from chicken meat and to maximize the discharge rate, and for this purpose, they are trying to modernize traditional methods. In this context, an innovative solution has been developed in this study to minimize the amount of blood remaining in the animal's body, to catch the highest blood discharge rate in drumsticks like in the breast meat, and to prevent blood retention in the drumstick at the maximum level. The effect of this innovative process step on the physical, chemical and microbiological quality of chicken drumstick meat was investigated. As a result of the analysis, the fact that an extra cut on the drumstick did not have a negative effect on the nutritional quality of chicken meat. It has been determined as a result of the analyzes that it has positive effects on some physicochemical properties as well as not having negative effects as has been stated above.

As a small addition to the process, preliminary tests have shown that an additional cut in the cartilage tissue of the chicken legs does not have a negative effect on the physical, chemical, microbiological and nutritional quality characteristics of the chicken meat, and the results of the analysis have objectively shown that the cut is an effective aid in removing the blood remaining in the tissue. Damaged tissues and bloody appearance are among the major problems that cause meat to drop from A to B quality. It is predicted that this innovative step will find a wide field of application, considering that it is an important factor in the classification of the meat as A quality, while the sensory and visual quality of the meat is improved as a result of the effective removal of the blood remaining in the tissues.
